# Host and environmental predictors of exhaled breath temperature in the elderly

**DOI:** 10.1186/1471-2458-13-1226

**Published:** 2013-12-23

**Authors:** Esmée Bijnens, Nicky Pieters, Harrie Dewitte, Bianca Cox, Bram G Janssen, Nelly Saenen, Evi Dons, Maurice P Zeegers, Luc Int Panis, Tim S Nawrot

**Affiliations:** 1 Centre for Environmental Sciences, Hasselt University, Agoralaan Building D, Diepenbeek 3590, Belgium; 2 Department of Public Health, Leuven University (KU Leuven), Leuven, Belgium; 3 Primary health care center GVHV, Genk, Belgium; 4 VITO, Flemish Institute for Technological Research, Mol, Belgium; 5 Transportation Research Institute, Hasselt University, Agoralaan Building D, Diepenbeek 3590, Belgium; 6 Department of Complex Genetics, NUTRIM School for Nutrition, Toxicology and Metabolism, Maastricht University, Maastricht, The Netherlands

## Abstract

**Background:**

Exhaled breath temperature has been suggested as a new method to detect and monitor pathological processes in the respiratory system. The putative mechanism of this approach is based upon changes in the blood flow. So far potential factors that influence breath temperature have not been studied in the general population.

**Methods:**

The exhaled breath temperature was measured in 151 healthy non-smoking elderly (aged: 60–80 years) at room temperature with the X-halo device with an accuracy of 0.03°C. We related exhaled breath temperature by use of regression models with potential predictors including: host factors (sex, age) and environmental factors (BMI, physical activity, and traffic indicators).

**Results:**

Exhaled breath temperature was lower in women than in men and was inversely associated with age, physical activity. BMI and daily average ambient temperature were positively associated with exhaled breath temperature. Independent of the aforementioned covariates, exhaled breath temperature was significantly associated with several traffic indicators. Residential proximity to major road was inversely associated with exhaled breath temperature: doubling the distance to the nearest major intense road was observed a decrease of 0.17°C (95% CI: -0.33 to -0.01; p = 0.036).

**Conclusions:**

Exhaled breath temperature has been suggested as a noninvasive method for the evaluation of airway inflammation. We provide evidence that several factors known to be involved in proinflammatory conditions including BMI, physical activity and residential proximity to traffic affect exhaled breath temperature. In addition, we identified potential confounders that should be taken into account in clinical and epidemiological studies on exhaled breath temperature including sex, age, and ambient temperature.

## Background

Exhaled breath temperature has been proposed to reflect airway inflammation as a positive relationship was observed between exhaled breath temperature and bronchial blood flow and exhaled nitric oxide (eNO) in asthmatic patients [1]. In other studies of asthmatic subjects, exhaled breath temperature was found to correlate with both eNO and sputum eosinophils [2] while treatment with inhaled corticosteroids led to a decline in the temperature of the exhaled breath and eosinophils and improved expiratory flow rates [3]. Moreover, the distinct difference in exhaled breath temperature reported between asthmatics and healthy subjects, while axillary and otic temperatures were the same, suggests that the increase in temperature of the expired breath is an unique indicator for the inflammatory status of the airways [4]. But even though exhaled breath temperature is elevated in persons with asthma, other factors might influence lung inflammation and therefore potentially influence exhaled breath temperature. The importance of other determinants of exhaled breath temperature have not yet been studied in the general population. Indeed, so far, all studies assessing exhaled breath temperature were applied in clinical samples of patients with asthma, chronic obstructive pulmonary disease (COPD) or in samples of healthy subjects and have often been limited in sample size. Studies in the population at large are therefore necessary in order to identify the factors that influence exhaled breath temperature. We measured exhaled breath temperature among 151 persons aged 60 to 80, to study the influence of host factors including gender and age as well as environmental factors such as physical activity, blood pressure, cholesterol levels and residential proximity to major roads as a proxy for exposure to traffic-related air pollution including ultra fine particulates [5-7].

## Methods

### Study population and sample collection

The total population (n = 3069) of the general medical practice in Genk (Belgium) is registered in the framework of a registration network for family practices in Flanders (INTEGO) [8]. The study area is representative of the total population. Non-smoking men and women, aged 60 to 80 years, with no acute infection at enrollment and no history of malignancies, were selected in the southern region of Genk [9]. Of those that were eligible, 188 were recruited by the general practitioner which resulted in a participation rate of 92%. Of these, 37 (19%) had no valid exhaled breath temperature measurement due their inability to breathe at a constant flow until a steady temperature was achieved. Characteristics of the persons with no valid measurement of exhaled breath temperature (n = 37) were compared with the final study population of 151 participants (Additional file 1: Table S1). Informed consent was obtained from all participants and the study was approved by the Ethical Committee of the East-Limburg Hospital (ZOL).

### Questionnaire data

Questionnaires were administered through a face-to-face interview to assess lifestyle, profession, education, past smoking status, as well as data on age, weight and gender. Most of the elderly in this study group were retired. Based on the information concerning last profession, we defined two categories blue versus white collar workers. Family income was given as net monthly overall family income and subdivided into low (<1500€), medium (1500€ - 3000€) and high (>3000€) family income. Education was coded as low (primary school), medium (high school) and high (university). Self-reported physical activity was assessed by the number of times a week a person was engaged in physical activity more than 30 minutes (including walking, cycling, gardening, sports,…). We gathered information on current and past use of medication from medical records of the general practice.

### Meteorological variables

We obtained daily average temperatures and daily average relative humidity from the Belgian Royal Meteorological Institute. The region of Flanders is very uniform for temperature and humidity, since both altitudinal and latitudinal gradients are extremely small: elevations range from 0 to 200 m above sea level and the distance between the northernmost and southernmost part is only 100 km. Therefore, we used temperature and humidity data from the central and representative station in Uccle (Brussels).

### Residential traffic-related pollution exposures

Residential addresses of all study participants were geocoded and distances to the nearest major road and traffic density were determined using standard Geographic information system (GIS) functions. According to functional class, we distinguished two types of roads: major roads and freeways. Major road was defined twice, once as all major roads, and once as a road with annual average daily traffic larger than 10,000 vehicles/day (major intense road). Major roads and major intense roads were highly correlated (R^2^ = 24%, p = 0.005). Additionally, we determined the direction of the major roads relative to the location of the participant. Traffic density was calculated as the sum of the modeled traffic intensity multiplied by road length for all roads in buffers with different radii (75 m, 200 m, 300 m, 500 m and 1 km). The selection of buffer sizes was done carefully and was mainly based on known dispersion patterns for traffic pollutants. All GIS analyses were conducted in ArcGIS 9.3.

### Clinical measurements

Exhaled breath temperature was measured with the X-halo device (Delmedica, Singapore) with an accuracy of 0.03°C. The patient inhaled through the nose and exhaled through the mouth directly into the X-halo device during 1 to 5 minutes until a stable temperature was reached. Expiratory air at the mouth is little influenced by ventilation rate [10] and pattern [1,10-12]. Subjects exhaled into an isolated chamber until the thermal sink placed inside was warmed up to the level of their exhaled breath temperature. This approach has the advantage of making exhaled breath temperature assessment less dependent on ambient factors and on the breathing pattern [3]. The tests were carried out in a room where the ambient temperature was between 18°C and 23°C and the ambient humidity between 28% and 50%. After the participants had rested for 5 minutes, the heart rate and blood pressure were stable and 7 consecutive blood pressure readings were taken by an automatic device (STABIL-O-GRAPH, Germany) according to the guidelines of the European Society of Hypertension [13]. Kralimarkova et al. [14] observed an effect of food ingestion on the exhaled breath temperature over 60 minutes. In our study all subjects had no food intake the 60 minutes prior to the measurement.

### Biochemistry

Blood samples were collected in Vacutainer® Plus Plastic K2EDTA Tubes (BD, Franklin Lakes, NJ, USA) and analyzed in the clinical laboratory of the regional hospital ZOL in Genk. Blood cell counts and differential leukocyte counts were determined using an automated cell counter with flow differential (Cell Dyn 3500, Abbott Diagnostics, Abott Park, IL, USA). Blood glucose levels, total cholesterol, high-density lipoprotein (HDL) cholesterol, low-density lipoprotein (LDL) cholesterol and triglycerides were measured according to standard clinical procedures.

### Statistical analysis

We used SAS software version 9.2 (SAS Institute Inc, Cary, NC). To study the relation between exhaled breath temperature and host and environmental parameters, we applied multiple linear regression. Parameters considered for entry in the model (p ≤ 0.15) by a stepwise regression were sex, age, family income, education, blue/white collar worker, past smoking status, physical activity, body mass index (BMI), glucose level, total cholesterol, HDL/LDL cholesterol, systolic and diastolic blood pressure, statins, anti-hypertensive medication, acetylsalicylic acid, COPD, asthma and daily average ambient temperature and daily average relative humidity. Other medications besides the ones mentioned above were not examined due to the small number of subjects reporting their use. Only determinants of exhaled breath temperature remained (p ≤ 0.10). Distance to roads were log transformed. The relation between exhaled breath temperature and traffic indicators was analyzed by the use of a multiple regression model corrected with the aforementioned significant host determinants of exhaled breath temperature. However asthma and COPD were no significant determinants they were forced into the regression model.

## Results

The characteristics of the 151 study participants are listed in Table 1. Exhaled breath temperature averaged (± SD) 33.2 ± 1.3°C. The mean (± SD) distance from the residence of the participants to the freeway was 3538 ± 1334 m (median, 3748 m) and the mean (± SD) distance from the residence to a major road was 644 ± 458 m (median, 539 m) (Table 2).

**Table 1:** Study population characteristics

**Characteristics**	**Men (n = 68)**	**Women (n = 83)**
Exhaled breath temperature, °C	33.5 (1.2)	33.0 (1.4)
Age, years	70.5 (5.0)	71.1 (4.3)
Family income		
-low	26 (38.8%)	43 (51.8%)
-medium	39 (58.2%)	39 (47%)
-high	2 (3.0%)	1 (1.2%)
Education		
-low	24 (35.8%)	36 (43.4%)
-medium	29 (43.3%)	31 (37.4%)
-high	14 (20.9%)	16 (19.3%)
White collar worker	29 (42%)	61 (73.5%)
Former smokers	52 (77.6%)	29 (34.9%)
Physical activity, times per week during 30 min	4.1 (2.7)	3.3 (2.5)
BMI, kg/m^2^	27.4 (3.6)	27.8 (5.1)
Glucose, mg/dl	108.6 (39.4)	99.1 (23.2)
Total cholesterol, mg/dl	193.6 (35.6)	207.1 (39.4)
HDL/LDL cholesterol, mg/dl	0.49 (0.19)	0.63 (0.33)
Systolic BP, mmHg	146.6 (16.1)	144.6 (20.2)
Diastolic BP, mmHg	89.3 (9.9)	85.8 (11.6)
Statins	36 (52.9%)	37 (44.6%)
Anti-hypertensive medication	39 (57.4%)	49 (59.7%)
Acetylsalicylic acid	38 (55.9%)	30 (36.6%)
COPD	10 (14.7%)	3 (3.6%)
Asthma	4 (5.9%)	2 (2.5%)

Data presented are means (SD) or number (%).

**Table 2:** Distribution of the traffic indicators

	**Percentile**					
**Traffic indicator**	**Mean ± SD**	**5th**	**25th**	**50th**	**75th**	**95th**
Distance: freeway, m	3538 ± 1334	810	2848	3748	4236	5292
Distance major road, m	644 ± 458	99	272	539	1011	1347
Distance major intense road, m	608 ± 515	67	228	518	1010	1239
Density: 200-m buffer, vehicles*km/day	2317 ± 3131	0	186	1130	2747	10338
Density: 300-m buffer, vehicles*km/day	6001 ± 6448	0	1716	3596	6890	19937
Density: 500-m buffer, vehicles*km/day	16973 ± 14057	1141	6465	10143	28241	42636
Density: 1000-m buffer, vehicles*km/day	65695 ± 31910	23160	34747	67948	92375	112646

Stepwise regression included sex, age, family income, education, blue/white collar worker, past smoking status, physical activity, BMI, glucose, total cholesterol, HDL/LDL cholesterol, systolic and diastolic blood pressure, statins, anti-hypertensive medication, acetylsalicylic acid, COPD, asthma and average ambient daily temperature and daily average relative humidity. Results of the stepwise regression showed that exhaled breath temperature is lower in women than in men, is negatively associated with age and physical activity, and positively associated with BMI and daily average ambient temperature, explaining 5.73% (p = 0.0006), 6.45% (p = 0.0020), 6.19% (p = 0.0110), 3.98% (p = 0.048), and 2.22% (p = 0.017) of its variance, respectively (Table 3).

**Table 3:** Correlates of exhaled breath temperature (°C) in multiple regression model

	**Adjusted regression coefficients**	**95% CI**			**P-value**	**Partial R**^**2**^
Age, +1 year	-0.069°C	-0.112	to	-0.026	0.0020	6.45%
Women	-0.714°C	-1.110	to	-0.319	0.0006	5.73%
Physical activity, + 30 min/week	-0.104°C	-0.184	to	-0.025	0.011	6.19%
BMI, + 1 kg/m^2^	0.048°C	0.001	to	0.096	0.048	3.98%
Daily average ambient temperature, +1°C	0.088°C	0.018	to	0.158	0.017	2.22%

Predictors identified from a stepwise regression model with parameters included; sex, age, family income, education, blue/white collar worker, past smoking status, regular physical activity, BMI, glucose, total cholesterol, HDL/LDL cholesterol, systolic and diastolic blood pressure, statins, anti-hypertensive medication, acetylsalicylic acid, COPD, asthma, daily average ambient temperature and daily average relative humidity.

**Figure 1:**
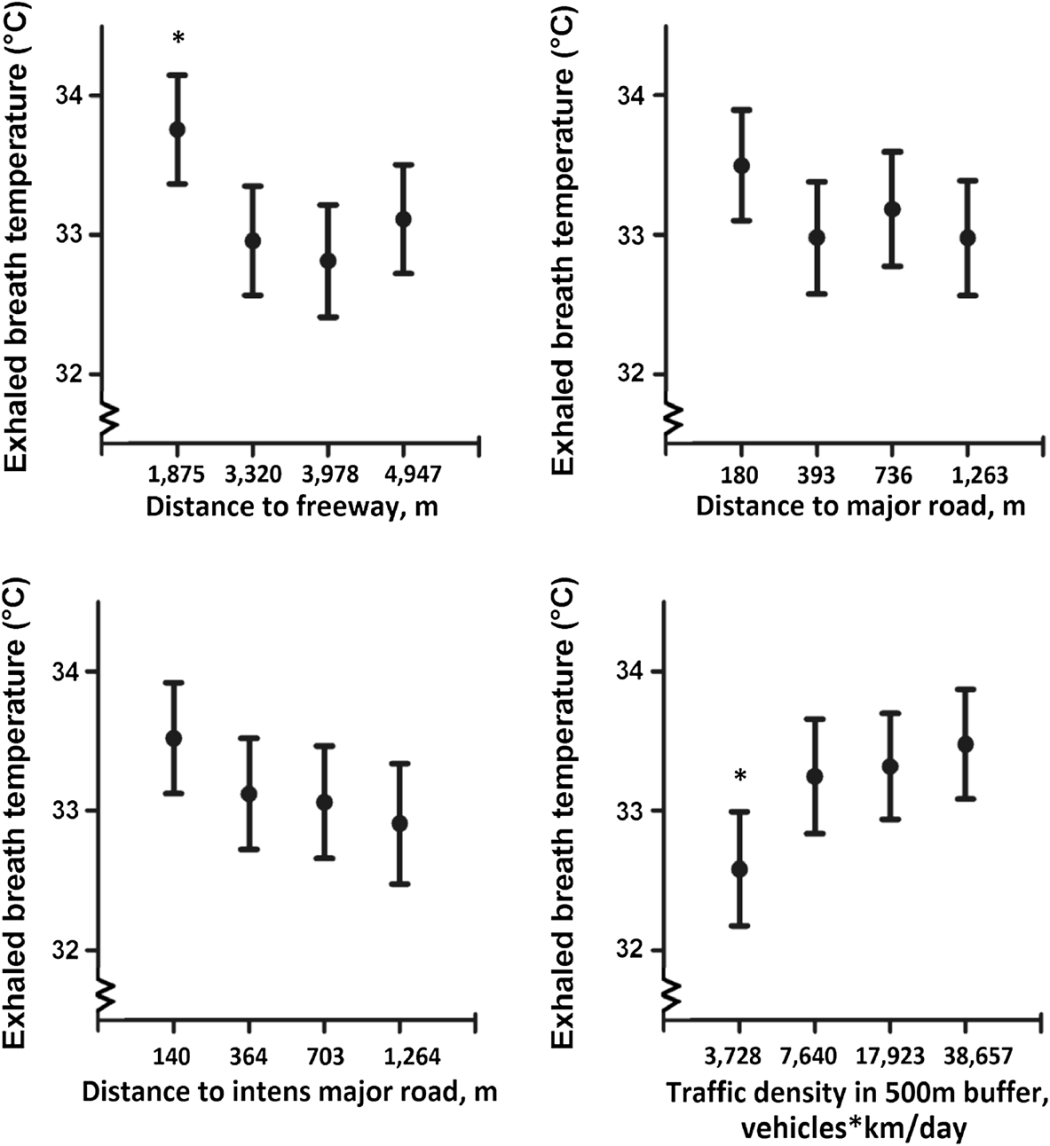
**Exhaled breath temperature as a function of residential exposure to traffic.** Mean exhaled breath temperature given in quartiles of traffic indicators, adjusted for gender, age, physical activity, BMI, daily average ambient temperature, asthma and COPD. Vertical lines denote 95% confidence intervals. *indicates quartile of exposure that is significant different (p < 0.05) from the other exposure quartiles.

Independent of the aforementioned covariates, several geocoded variables reflecting traffic related exposure were significantly associated with exhaled breath temperature (Figure 1). Exhaled breath temperature was significant lower for elderly when doubling the distance from important roads such as freeways (β = -0.23°C, 95% CI: -0.42°C to -0.03°C; p = 0.028), major roads (β = -0.18°C, 95% CI: -0.35°C to -0.001°C; p = 0.050) and major intense roads with a traffic intensity of more than 10 000 vehicles/day (β = -0.17°C, 95% CI: -0.33°C to -0.01°C; p = 0.036). Wind direction between residence and road had no major role as it was not related with exhaled breath temperature either directly (P = 0.62) nor in interaction with distance to major road (P ≥ 0.68). Regarding traffic density, for each IQR increase of 21,775 vehicles*km/day within a 500 m radius of the participants’ home, we observed an increase in exhaled breath temperature of 0.37°C (Table 4). A sensitivity analysis with exclusion of 6 persons with asthma and 13 with COPD showed similar results (results not shown).

**Table 4:** **Estimated difference in exhaled breath temperature (°C) associated with an 100**% **increase in distance and an interquartile range increase in traffic density**

**Exposure (exposure contrasts)**	Adjusted regression coefficients*	**95% CI**			**P-value**
Distance: freeway, +100%	-0.23°C	-0.42	to	-0.03	0.028
Distance major road, +100%	-0.18°C	-0.35	to	-0.001	0.050
Distance major intense road, +100%	-0.17°C	-0.33	to	-0.01	0.036
Traffic density: 200-m buffer, +2 562 vehicles*km/day	0.13°C	-0.04	to	0.30	0.14
Traffic density: 300-m buffer, + 5 174 vehicles*km/day	0.14°C	-0.02	to	0.31	0.084
Traffic density: 500-m buffer, + 21 775 vehicles*km/day	0.37°C	0.06	to	0.68	0.021
Traffic density: 1000-m buffer, + 57 628 vehicles*km/day	0.21°C	-0.16	to	0.59	0.26

*Adjusted for gender, age, physical activity, BMI, and daily average ambient temperature as identified in Table 3 in addition asthma and COPD were forced in the regression models.

## Discussion

Exhaled breath temperature is one of the many characteristics of exhaled breath, the analysis of which is regarded as a promising non-invasive approach to respiratory and other diseases. Our population-based study found that exhaled breath temperature was higher in men compared with women. Furthermore, we showed that an increase in age was associated with a decrease in exhaled breath temperature within the study age range of 60–80 year olds. We identified BMI, physical activity and ambient temperature as important predictors of exhaled breath temperature in the elderly. In particular, BMI and ambient temperature were positively associated with exhaled breath temperature, while physical activity was inversely associated. Independent of the aforementioned host and environmental factors, several traffic related indicators were positively associated with exhaled breath temperature. Several factors identified in our study as predictors of exhaled breath temperature are known to produce free radicals and are involved in chronic tissue inflammation in the population at large [15-17]. The similarity of predictors of exhaled breath temperature seen in our study and eNO observed in other studies suggests that both markers reflect pathophysiological conditions. Exhaled NO has been considered as a non-invasive marker to measure inflammation and oxidative stress in the lung [18]. In pathological conditions inducing lung inflammation the inducible nitric oxide synthase enzyme (iNOS) generates extraordinarily high concentrations of NO when the body faces an inflammatory response by attracting macrophages that generate NO [19]. Due to proinflammatory changes in the structure and function of the lung blood vessels, they might widen, enlarge or proliferate supplying inflammatory cells in chronically inflamed tissues [20]. In this way airway inflammation might be responsible for increased bronchial blood flow and increased exhaled breath temperature.

Independent of the other factors, we found a higher exhaled breath temperature in elderly with a higher BMI compared to elderly with a low BMI. Our results with exhaled breath temperature are consistent with the previously published results on eNO in adults that indicate that obesity results in an upregulation of pulmonary inflammatory mechanisms [21,22]. A possible cause of this airway inflammation is the chronic low-grade systemic inflammation present in obese persons, evidenced by increased levels of proinflammatory molecules [23]. The increasing macrophage content in the adipose tissue of persons with a high BMI is possibly responsible for secretion of proinflammatory molecules, such as interleukin-6 (IL-6) [24,25].

Persons practicing regular physical activity had a lower exhaled breath temperature than persons with no regular activity, independent of age and other studied factors. In elderly, regular physical activity is associated with lower systemic inflammation and lower circulating levels of inflammatory biomolecules: C-reactive protein, fibrinogen, factor VIII activity, and white blood cell count and interleukin 6 [15,26-28]. Nicklas and colleagues [27] demonstrated that an intervention with an increase in physical activity in elderly reduces systemic concentrations of IL-6 even without any weight loss.

A possible explanation for the observed differences in exhaled breath temperature between men and women can be related to different hormone profiles. Mandhane et al. [29] observed an inverse association between estrogen levels and exhaled nitric oxide, a marker of airway inflammation, during normal menstrual cycle.

Moreover, exhaled breath temperature was shown to be clearly age dependent in the age range of 60–80 year olds and decreased with ageing. Other studies found no association between exhaled breath temperature and age in children (age range: 9–11 years, n = 60) [30] and the exhaled air temperature was not statistically different in a relatively underpowered study of younger (20 ± 6 years, n = 8) compared to older (55 ± 9 years, n = 6) adults [31].

Additionally, we noticed that ambient day temperature is a significant determinant of exhaled breath temperature. Previous studies [30] observed a significant association between the exhaled breath temperature and room temperature, while this association was not seen by Popov and colleagues [4].

Possible effects of statins on lung function have been suggested. In the Normative Aging Study, statins attenuate the decline in lung function in elderly [32]. An experimental animal study observed that lovastatin significantly suppressed the PM_10_-induced lung inflammation [33]. A possible side effect of statin therapy is statin-induced interstitial lung disease [34]. In our study we did not observe an effect of statins on exhaled breath temperature.

Finally, exhaled breath temperature was significantly related to traffic indicators. The exhaled breath temperature increases with freeway and major road proximity and with increasing traffic density. Our findings on traffic indicators and exhaled breath temperature are also biologically robust because they are consistent with a large body of data on the adverse effects of air pollutants on the lung and cardiovascular system [35-39]. Ultrafine particles represent a toxicologically important fraction of vehicle emissions, although they hardly affect the mass concentrations of particulates (PM_10_ or PM_2.5_). Combustion-generated ultrafine particles produced by diesel engines are believed to be, at least partly, responsible for the cardiopulmonary effects of outdoor urban air pollution, possibly through proinflammatory effects [40-43]. The effect on the exhaled breath temperature induced by a doubling in distance to a major intense road is equivalent to the effect of 3.5 kg/m^2^ decrease in BMI.

The present study should be interpreted within the context of its limitations. First, we do not have intermediate markers of systemic inflammation or eNO measurements. Second, the cross-sectional nature of the study limits the extent of causal inferences that can be drawn from our data. Nevertheless, these are the first data for a general sample of elderly. We achieved a very high participation rate. Our results are unlikely to be biased by selection, first we achieved a very high participations rate and those that had no valid temperature measurement (n = 37) did had similar sociodemographic characteristics such as sex, age, socio-economic variables, as well as past smoking status, physical activity, BMI. To date, most studies on exhaled breath temperature have been performed in asthmatic patients and have suggested the utility of this approach to assess non-invasively changes in the degree of airway inflammation. In our population of elderly, asthma and COPD did not influence exhaled breath temperature upon the other factors identified, probably due to the limited number of persons with these pathophysiological conditions (n = 6 and n = 13 respectively).

## Conclusion

We identified at the population level several determinants of exhaled breath temperature. In further research on exhaled breath temperature it is important that these factors are taken into account. Exhaled breath temperature might be an interesting biomarker or clinical tool for monitoring proinflammatory conditions induced by environmental factors. The clinical significance in the prediction of outcomes within the healthy population must be further elucidated.

## Abbreviations

BMI: Body mass index; C: Celsius; CI: Confidence interval; COPD: Chronic obstructive pulmonary disease; EDTA: Ethylenediaminetetraacetic acid; eNO: Exhaled nitric oxide; GIS: Geographic information system; HDL: High-density lipoprotein; IL-6: Interleukin-6; iNOS: Inducible nitric oxide synthase enzyme; IQR: Inter quartile range; K2EDTA: Dipotassium ethylenediaminetetraacetic acid; km: Kilometer; LDL: Low-density lipoprotein; m: Meter; NO: Nitric oxide; SD: Standard deviation; ZOL: East-Limburg Hopital.

## Competing interests

The authors declare that they have no competing interests.

## Authors’ contributions

TSN designed the study together with EB, NP, and HD. EB did the statistical analysis with help of BC and MZ. Field work was done by NP, BGJ, NS. Traffic related exposure indexes were constructed by ED and LIP. EB and NP wrote the first draft of help of TSN. TSN is the guarantor of the content. All authors have read and approved the submission of the manuscript and accept responsibility for the contents.

## Pre-publication history

The pre-publication history for this paper can be accessed here:

http://www.biomedcentral.com/1471-2458/13/1226/prepub

## Supplementary Material

Additional file 1: Table S1 Characteristics of persons with no valid measurement. Click here for file
