# Supplementary material for: Host and environmental predictors of exhaled breath temperature in the elderly
Source: BMC Public Health. 2013 Dec 23;13:1226. doi: 10.1186/1471-2458-13-1226 (PMC3890614; doi:10.1186/1471-2458-13-1226)
Supplement: Additional file 1: Table S1 — Characteristics of persons with no valid measurement. [file 1471-2458-13-1226-S1.doc]

**Supplement table 1 Characteristics of persons with no valid measurement**

|  |  |  |
| --- | --- | --- |
| **Characteristics** | **No valid measurement (n=37)** | **Final study population (n=151)** |
|  |  |  |
| Sex, man | 18 (56.3%) | 68 (45.0%) |
| Age, years | 70.7 (4.4) | 70.8 (4.6) |
| Family income |  |  |
| -low | 14 (45.2%) | 69 (46.0%) |
| -medium | 17 (54.8%) | 78 (52.0%) |
| -high | 0 (0%) | 3 (2.0%) |
| Education |  |  |
| -low | 13 (41.9%) | 60 (40.0%) |
| -medium | 9 (29.0%) | 60 (40.0%) |
| -high | 9 (29.0%) | 30 (20.0%) |
| Former smokers, yes/no | 17 (54.8%) | 81 (54.0%) |
| Physical activity, times per week during 30min | 3.9 (2.7) | 3.6 (2.6) |
| BMI, kg/m^2^ | 26.8 (4.1) | 27.6 (4.5) |
|  |  |  |

Data presented are means (SD) or number (%)
